# Thermally and photoinduced structural and chemical changes of a silver nanocube array on Au(111)

**DOI:** 10.1039/d1ra00830g

**Published:** 2021-04-28

**Authors:** Takeru Iwahori, Ayana Mizuno, Atsushi Ono, Yoichi Uehara, Satoshi Katano

**Affiliations:** Research Institute of Electrical Communication, Tohoku University 2-1-1 Katahira, Aoba-ku Sendai 980-8577 Japan skatano@riec.tohoku.ac.jp +81-22-217-5498; Graduate School of Integrated Science and Technology, Shizuoka University Hamamatsu 432-8561 Japan; Research Institute of Electronics, Shizuoka University Hamamatsu 432-8011 Japan

## Abstract

We have investigated the thermally and photoinduced structural and chemical changes of a polyvinylpyrrolidone (PVP)-covered silver nanocube (AgNC) array on Au(111). The Langmuir–Blodgett (LB) method was utilized to fabricate the highly ordered array of the AgNC monolayer on Au(111). In the Raman spectra obtained at room temperature, sharp vibrational peaks were observed owing to the surface-enhanced Raman scattering (SERS) effect of AgNCs. When AgNCs were annealed, their corners became rounded, followed by their height decrease and lateral expansion. Simultaneously, PVP decomposed into nanocarbons, which were eliminated from the gap between AgNCs. Further annealing AgNCs/Au(111) resulted in obvious decreases in Raman signal intensity and AgNC height due to the sintering of AgNCs. We also confirmed the photochemical transformation of PVP to nanocarbons without the deformation of AgNCs when an intense laser was irradiated on the AgNC surface.

## Abstract 2

We have investigated the thermally and photoinduced structural and chemical changes of a polyvinylpyrrolidone (PVP)-covered silver nanocube (AgNC) array on Au(111).

## Graphical abstract

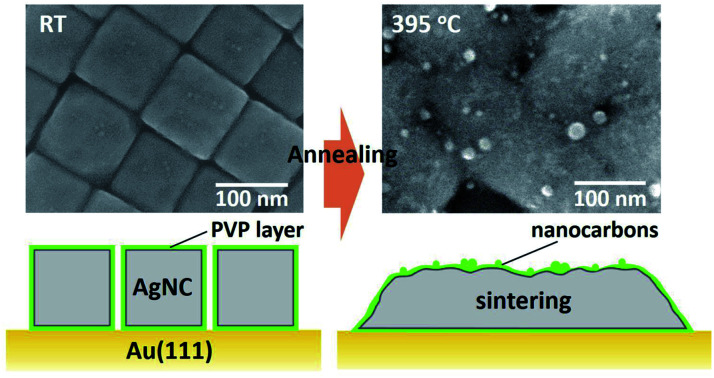

## 1. Introduction

Metal nanoparticles (NPs) forming an array on a substrate have been attracting much attention in relation to technological applications such as sensing, optoelectonics and catalyses.^1–3^ So far, various metals and shapes of NPs have been used to fabricate NP arrays. For example, NP arrays made of gold (AuNP)^4–7^ and silver (AgNP)^8–13^ are representative and have been extensively studied. The key factors in the determination of the physical and chemical properties of metal NP arrays are their shape, size and arrangement. Microscopic structures of NP arrays obviously affect the surface-enhanced Raman scattering (SERS) effect, localized surface plasmon resonance (LSPR) and reaction activity.^14–17^

Many studies have been conducted to control the shape, size of individual NPs and arrangement of NP arrays through thermal treatment. Asoro *et al.* showed that isolated AgNPs sublimate uniformly upon heating without transforming to the liquid phase.^18^ Generally, the sublimation temperature increases as the size of NPs increases. For example, 5 nm AgNPs sublimate at around 500 °C, whereas 30 nm AgNPs sublimate at around 675 °C.^18^ On the other hand, it is known that thermal changes in the morphology and arrangement of NP arrays occur at a temperature lower than that in the case of isolated NPs.^4,5,7,19^ For example, Zhang *et al.* demonstrated that heating treatment causes the aggregation of AuNPs at 100 °C and the coalescence of AuNPs at 140 °C.^4^ Laser-induced shape conversions have been also reported for Au nanorods, Ag nanoplates and AgNPs in colloidal suspensions.^20,21^ Thermal and photoinduced structural changes of NPs are often accompanied by a shift in LSPR wavelength and are proposed to control the plasmon properties of NPs.^4,5,7,19–24^

Recently, a silver nanocube (AgNC) has drawn much attention since it has a higher electric field enhancement^25–31^ than and a superior catalytic activity to AgNP of spherical shape.^32,33^ Unique nanoscale properties, such as single-molecule detection by the SERS effect,^25^ superior catalytic activity on (100) facets^32,33^ and plasmonic chemical reactions,^34–36^ and brightening of an organic light-emitting device,^37^ have been realized by utilizing AgNCs. Similarly to the spherical NP system, the shape and size of individual AgNCs can be controlled by heating and light irradiation. Vijayan and Aindow heated isolated AgNCs and examined the temporal change of the AgNC structure.^38^ They revealed that AgNCs remained intact below 400 °C, whereas they were sublimated and became spherical at around 760 °C. Several research groups demonstrated that AgNCs behave as quasiliquids above 500 °C and (110) facets are preferentially formed by surface relaxation during sublimation.^39,40^ As described above, although shape changes of isolated AgNCs have been well studied, those of AgNCs in an array have not been reported. Several research groups pointed out that unique characteristics, such as the enhanced SERS effect^26,29^ and selective excitation of localized surface plasmon (LSP) modes,^27^ appear in the AgNC array. Thus, it is highly expected that the optical properties of AgNC arrays can be tuned by changing the size and alignment of AgNCs. To the best of our knowledge, there has been no report on the structural changes of the AgNC array upon heating or laser irradiation.

In this study, we have investigated the structural change of an AgNC array upon heating and laser irradiation, and also characterized the simultaneous changes in the chemical states of polyvinylpyrrolidone (PVP) covering the AgNCs. About 100nm-sized AgNCs covered with PVP were synthesized by the polyol method. The Langmuir–Blodgett (LB) method was utilized to fabricate a monolayer array of AgNCs on Au(111). The AgNC array on Au(111) was heated in an ultrahigh vacuum (UHV) chamber and irradiated with a laser in atmosphere. Then, the AgNC shapes and arrangements were evaluated by scanning electron microscopy (SEM) and atomic force microscopy (AFM). Furthermore, we used Raman spectroscopy to elucidate the chemical state of PVP before and after heating and laser irradiation.

## 2. Experimental method

100 nm-sized AgNCs were synthesized by the standard technique of the polyol method with reference to previous studies.^3^ After 1 mL of 3 mM HCl was added in 5 mL of ethylene glycol (EG) heated to 130 °C, 3 mL each of 94 mM AgNO_3_/EG solution and 147 mM PVP (Sigma-Aldrich; average molecular weight, ∼55 000)/EG was dropped into with a flow rate of 0.75 mL min^−1^. The AgNCs were synthesized by keeping the temperature at 130 °C for 20 hours. The synthesized solution was purified by centrifuging at 2000 × *g* for 20 minutes. It is known that PVP adsorbed selectively to the (100) faces, leading to the growth of cubically shaped Ag nanoparticle. Therefore, each AgNC facet was fully covered with PVP immediately after the AgNC was synthesized. It is important to use AgNCs with a uniform size since the size of AgNC affect the SERS activity. For this reason, we used 100 nm-sized AgNCs because we succeeded in producing AgNC of this size with good reproducibility. In addition, we chose the size of AgNC in terms of the resonance wavelength of AgNC's LSP mode. The Au(111) substrate was obtained by evaporating Au (purity, 99.99%) on a freshly cleaved mica surface under vacuum (3 × 10^−4^ Pa), followed by annealing at 500 °C.^41–43^ The 2D array of AgNCs was fabricated on Au(111) by the LB method.^27^ We evaluated the shape and array structures of AgNCs by SEM (Hitachi High-tech, SU8000) and AFM (SII, SPI3700, contact mode). Raman spectroscopy (JASCO, NRS5100) was performed at room temperature under an atmosphersic condition using a laser of 532 nm wavelength (spot diameter, 1 μm, power, 0.01 mW), a 600 lines per mm diffraction grating, and a ×100 objective (N.A. = 0.90). AgNCs/Au(111) was subsequently heated from room temperature to 111, 179, 222, 265, 395 and 482 °C in an UHV chamber (1 × 10^−7^ Pa). The sample was heated by resistive heating. After reaching the set temperatures, AgNCs/Au(111) was retained at the temperature for 20 min, followed by cooling to room temperature. After the annealing treatment, AgNCs/Au(111) was removed into the atmosphere, followed by SEM, AFM and Raman measurements. Photochemical reactions were induced at room temperature in atmosphere by laser irradiation to AgNCs/Au(111) using the laser equipped in the Raman spectroscope. The weakness of silver is low resistance to oxidation and sulfidation, which causes instability in the various physical and chemical properties.^44^ However, it has been reported that strongly adsorbed ligand molecules on metal are known to provide long-term stability to the particles, protecting them from oxidation.^45^ In this study, the surfaces of AgNC are always covered with PVP and/or decomposed products. Thus, we presume that contaminations of AgNC in air is sufficiently suppressed.

## 3. Results and discussion

**Fig. 1:**
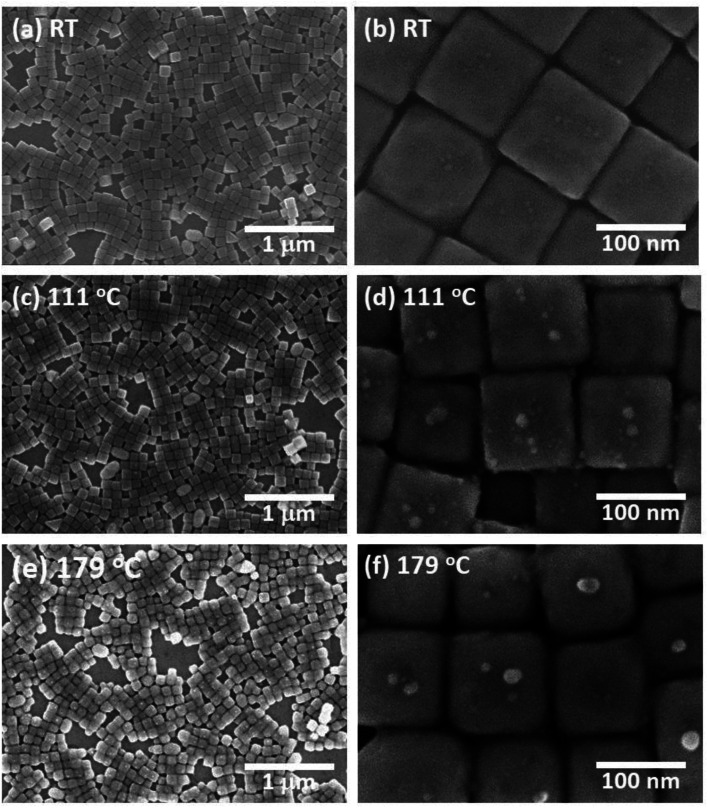
(a) SEM image of PVP/AgNC/Au(111) at room temperature (RT) fabricated by the LB method. Enlarged SEM image is shown in (b). SEM images of PVP/AgNC/Au (111) obtained after annealing at (c) 111 °C and (e) 179 °C. Enlarged SEM images obtained after annealing at 111 °C and 179 °C are shown in (d) and (f). Acceleration voltage (*V*_Acc_) was set at 10 kV in all images.

**Fig. 2:**
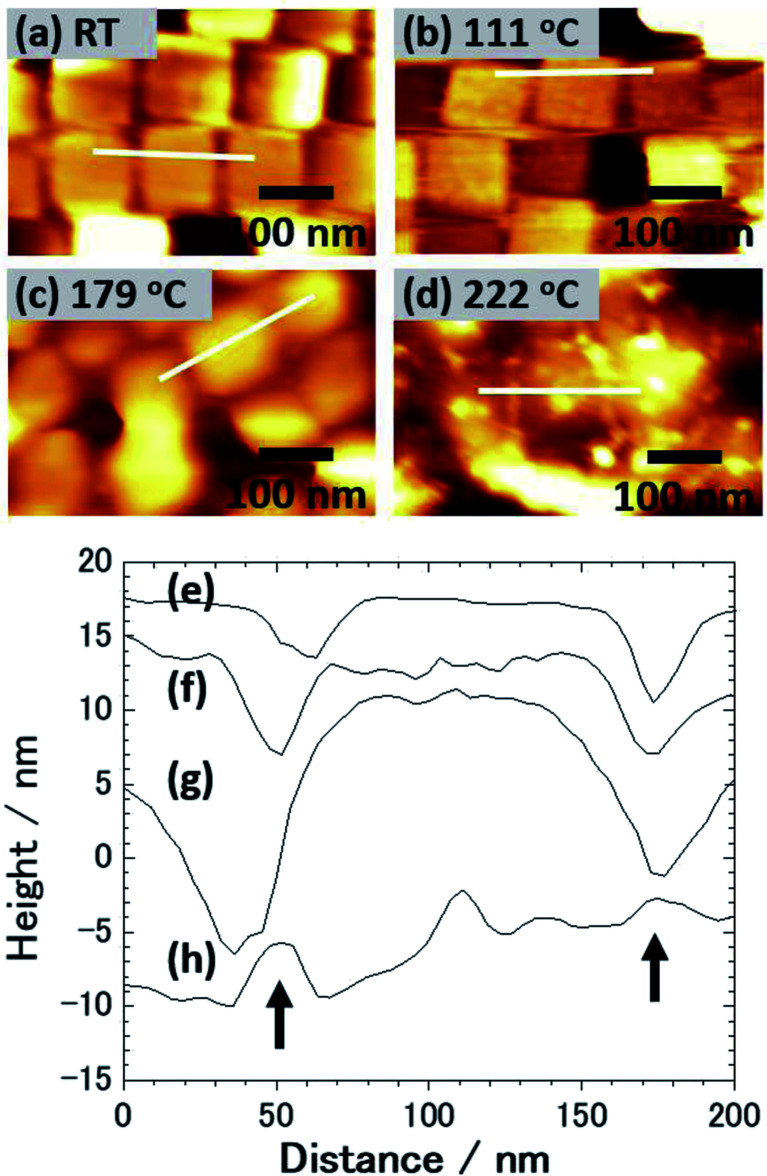
AFM images of PVP/AgNC/Au(111) obtained at (a) RT and after annealing at (b) 111 °C, (c) 179 °C and (d) 222 °C. (e–h) Height profiles taken along the lines shown in (a–d), respectively. Arrows in (h) indicate the positions that originally corresponded to the centers of gaps between AgNCs.

**Fig. 3:**
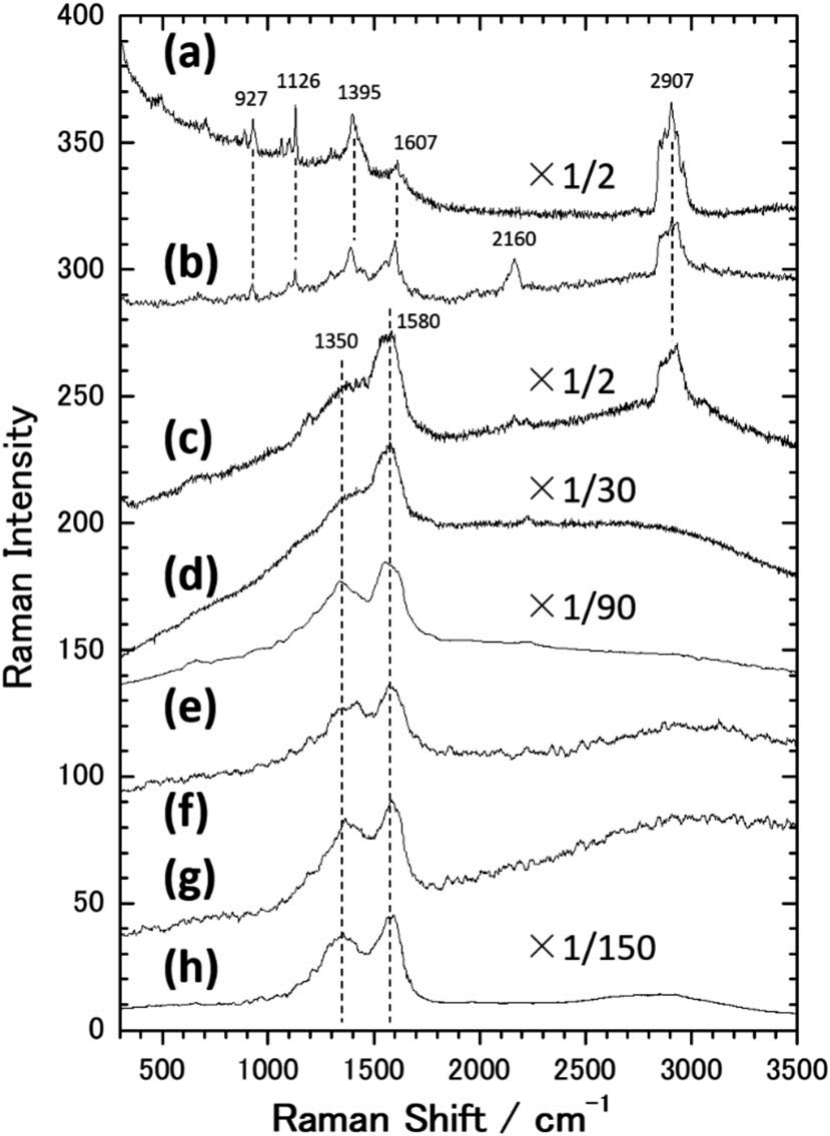
Raman spectra of PVP/AgNC/Au(111) obtained at (a) RT and after annealing at (b) 111 °C, (c) 179 °C, (d) 222 °C, (e) 265 °C, (f) 395 °C and (g) 482 °C. (h) Raman spectrum obtained after the irradiation of intense laser (*λ* = 532 nm, 4 mW μm^−2^) to PVP/AgNC/Au (111) for 4 s (2 s × 2 times). All of the spectra were obtained using the 532 nm laser with the power of 0.01 mW μm^−2^ and the exposure time of 10 s. Each spectrum was obtained after accumulating (a) 20 times, (b–e) 2 times and (f) 10 times. The adjacent means were taken for 0–10 points for each spectrum.

Fig. 1(a) shows an SEM image of the sample surface on which the AgNCs were accumulated by the LB method. The AgNCs are clearly resolved and cover the Au(111) surface with high density. Individual AgNCs are uniform in both size and shape, showing that chemical synthesis was performed with good controllability. Owing to the LB method, the AgNCs are aligned in the face-to-face configuration, forming a two-dimensional monolayer. The enlarged SEM image shown in Fig. 1(b) shows that the AgNCs are separated from each other with a gap distance of about 10 nm. The average size of AgNCs is estimated to be 103 nm. Fig. 2(a) and (e) show an AFM image and a height profile focused on several adjacent AgNCs aligned in the face-to-face configuration. The height analysis indicated that the root mean square (rms) roughness of the AgNC surface is 0.32 nm. This means that the facets of AgNC are almost atomically flat, as was pointed out in a previous paper.^27^Fig. 3(a) shows the Raman spectrum of PVP-covered AgNCs/Au(111) at room temperature. Peaks seen in this spectrum are assigned to the vibrational modes of PVP, which covers the AgNC's surfaces. This result ensures that PVP kept intact during the Raman measurement. Peaks between 2800 and 3000 cm^−1^ are assigned to stretching modes of CH and CH_2_. A peak at 1607 cm^−1^ is assigned to the stretching mode of C=O. Peaks between 800 and 1000 cm^−1^ are assigned to stretching modes of C–C and C–N. The details of the assignments of the vibrational peaks have been reported in the previous paper.^27^ Although the thickness of PVP is small (4–6 nm), the SERS effect of AgNCs enables us to observe vibrational peaks of PVP.^27^

Next, this sample was annealed in UHV to induce the structural change of AgNCs. Fig. 1(c) shows an SEM image of the AgNCs/Au(111) sample after annealing at 111 °C. An enlarged SEM image of the same surface is also shown in Fig. 1(d). The AgNC alignment remained unchanged after annealing, which implies that AgNCs do not diffuse or aggregate at 111 °C. To confirm the structures in detail, an AFM image and the height profile are presented in Fig. 2(b) and (f), respectively. As similarly observed in Fig. 2(a), adjacent AgNCs are aligned in the face-to-face configuration. The roughness of the AgNC surface is estimated to be 2 nm in the protruded areas, and the rms roughness is 0.68 nm, which suggests that the surface became slightly bumpy. Fig. 3(b) shows the Raman spectrum of PVP-covered AgNCs/Au(111) after annealing at 111 °C. In addition to the peaks assigned to vibrational modes of PVP, a broad peak newly appeared at 2160 cm^−1^. This peak consists of two peaks at 2157 and 2214 cm^−1^, which are assigned to the C≡N stretching modes.^46^ This is strong evidence of the ring opening of pyrrolidone. Therefore, we consider that PVP starts to decompose at 111 °C. We presume that such a chemical change of PVP accompanies its structural change, leading to the increase of roughness.

Fig. 1(e) shows an SEM image of AgNCs/Au(111) obtained after annealing at 179 °C. Individual AgNCs are still resolved and overall alignment is unchanged. The enlarged SEM is shown in Fig. 1(f). A careful structural analysis revealed that corners and edges of AgNCs became rounded, which was not mentioned in the previous paper.^38^ This structural change is more clearly seen in the AFM image (Fig. 2(c)) and height profile (Fig. 2(g)) of AgNCs/Au(111). Furthermore, it appears that the gaps between AgNCs became deeper after annealing at 179 °C. This is because the rounded corners and edges (or the large distance between the AgNCs) allow the AFM tip to follow deeply into the AgNC gaps. Fig. 3(c) shows the Raman spectrum of PVP-covered AgNCs/Au(111) obtained after annealing at 179 °C. Except for the peaks of CH and CH_2_ stretching modes between 2800 and 3000 cm^−1^, vibrational peaks assigned to PVP disappeared whereas intensely broad peaks appeared at 1350 and 1580 cm^−1^. According to the previous study,^47^ the peaks at 1350 and 1580 cm^−1^ can be assigned to D and G bands of nanocarbons, respectively. Thus, we considered that PVP was partially converted to nanocarbons having sp^2^ and sp^3^ carbons by annealing at 179 °C. The appearances of G and D bands in the Raman spectra obtained after the annealing is the proof for the formation of nanocarbons. However, Li and co-workers reported that in the case of sphere AgNP, the part of Ag atoms is possible to absorb into the carbonaceous cluster, forming the mixture of Ag and carbon.^48^ On the basis of Raman analysis, it is difficult to obtain the further evidence of the chemical reaction between nanocarbon and Ag.

**Fig. 4:**
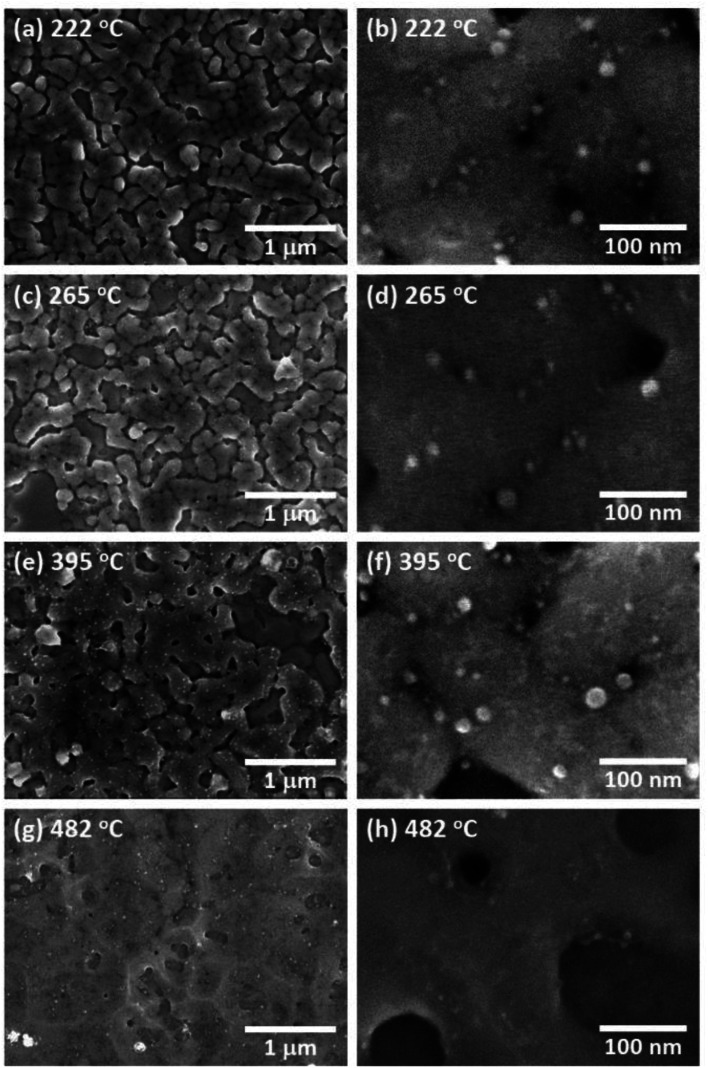
SEM images of PVP/AgNC/Au(111) obtained after annealing at (a) 222 °C, (c) 265 °C, (e) 395 °C and (g) 482 °C. Enlarged SEM images obtained after annealing at 222 °C, 265 °C, 395 °C and 482 °C are shown in (b), (d), (f) and (h) respectively. *V*_Acc_ was set at 10 kV in all images.

Fig. 4(a) shows an SEM image of AgNCs/Au(111) obtained after annealing at 222 °C. Individual AgNC edges became more ambiguous in most areas. This is clearly seen in the enlarged SEM image (Fig. 4(b)). Furthermore, it was found that nanoparticles with diameters of 5–20 nm were formed on and between AgNCs. The height profile (Fig. 2(h)) taken from the enlarged AFM image (Fig. 2(d)) clearly suggests that new nanoparticles having heights of a few nanometers formed on the AgNC surface and stuck in the gaps between AgNCs. Fig. 3(d) shows the Raman spectrum of PVP-covered AgNCs/Au(111) after annealing at 222 °C. The appearance of D and G bands indicates the presence of nanocarbons. It is noteworthy that CH and CH_2_ stretching peaks at 2800–3000 cm^−1^ completely disappeared. SEM observations revealed that the surface structures did not change significantly after annealing at 265 °C (Fig. 4(c) and (d)). The Raman spectrum (Fig. 3(e)) of this surface is similar to that in Fig. 3(d), whereas the intensities of both D and G bands increased. Further annealing of the AgNC/Au(111) sample at 395 °C resulted in the deformation of AgNCs, and hence, individual cubic structures were no longer preserved (Fig. 4(e) and (f)). On increasing the annealing temperature to 482 °C, we found that the AgNC grains drastically collapsed and expanded laterally, so that the area of the bare Au surface diminished (Fig. 4(g) and (h)). The Raman spectra obtained at 395 °C (Fig. 3(f)) and 485 °C (Fig. 3(g)) were similar to that in Fig. 3(e), whereas the intensities of both D and G bands drastically decreased.

**Fig. 5:**
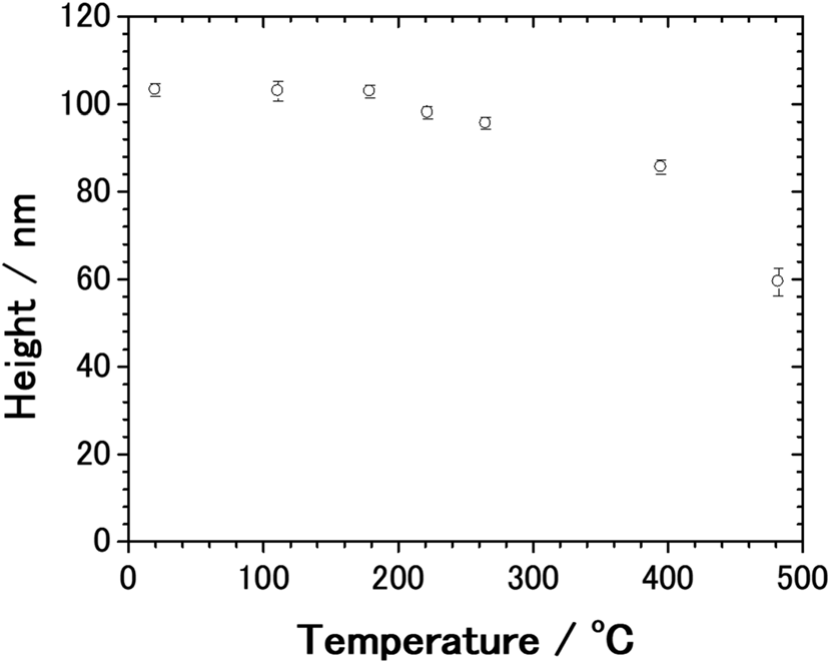
Plots of the average height of AgNCs against the annealing temperature. The average height of AgNCs was deduced by analyzing 20–30 AgNCs by AFM. The standard errors are indicated by the error bar.

From the AFM measurement, we found that the thermal deformation of AgNCs accompanies a change in AgNC height. In Fig. 5, the height of AgNC at each annealing temperature is indicated by the average value of 20–30 AgNCs, and the standard errors are indicated by the error bar. At room temperature, the average height of AgNCs is 103 nm, which corresponds to their side length. The AgNC height remained unchanged up to 179 °C, indicating that the cubic structure was preserved. Further increasing the annealing temperature resulted in a gradual decrease in average height, *i.e.*, 98.0 nm for 222 °C, 95.6 nm for 265 °C and 85.6 nm for 395 °C. When heated to 482 °C, the average height drastically dropped to 59.3 nm owing to the collapse of AgNCs. Note that owing to the ambiguity of AgNC edges above 222 °C, it is difficult to estimate the lateral length of individual AgNCs.

A previous thermal gravimetric analysis (TGA) of a pure PVP film in nitrogen atmosphere revealed a large reduction in mass due to decomposition and desorption of PVP at around 400 °C.^49–52^ Furthermore, Borodko and co-workers performed Raman spectroscopy and reported that the PVP covering on PtNP was converted to nanocarbon at 300–350 °C in the presence of oxygen or hydrogen.^46,53^ A comparison of the present result with those in the previous studies indicated that the decomposition of PVP was completed at a temperature higher than 265 °C.

**Fig. 6:**
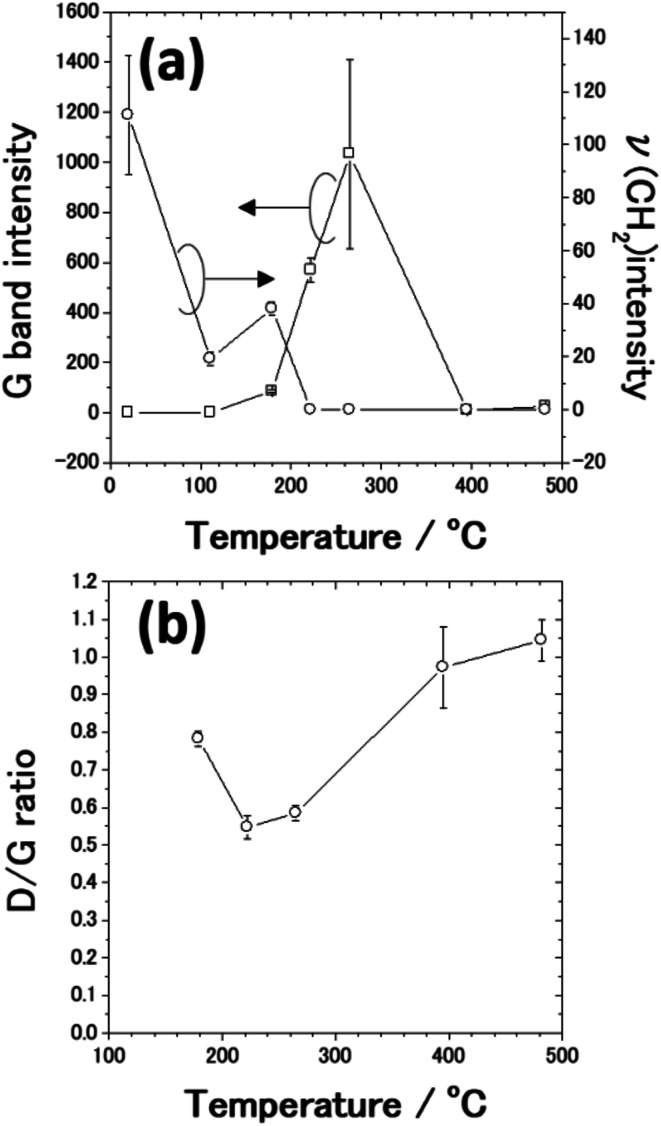
Plots of the Raman intensity of (a) G band and *ν*(CH_2_) peak of PVP and (b) the D/G ratio against the annealing temperature. The intensities of D and G bands were estimated by fitting the peaks using Gaussians. The *ν*(CH_2_) intensities were extracted from the peak at 2907 cm^−1^ in the Raman spectra. Each value was obtained from taking the average of 5–13 spectra. The standard errors are indicated by the error bar.

From SEM observations, we found that the AgNC gaps are difficult to identify when the annealing temperature exceeds 179 °C. Fig. 6(a) shows plots of the G band intensities as a function of the annealing temperature. G band intensities were estimated by fitting the peaks using Gaussians since the G band peak overlaps the D band peak. When PVP-covered AgNCs/Au(111) was annealed, the G band peak started to be observed at 179 °C. Fig. 6(a) also shows plots of the CH_2_ stretching peak intensity as a function of annealing temperature. The intensity of the *ν*(CH_2_) peak decreased from room temperature to 111 °C because of the opening of pyrrolidone rings. At 179 °C, the G band appeared and PVP started to be transformed to nanocarbons. Further decrease and vanishment of *ν*(CH_2_) peak intensity at 179 and 222 °C, respectively, are associated with the decomposition of PVP. In contrast, the G band peak intensity increased owing to the formation of nanocarbons up to 265 °C. However, when the sample was annealed at 395 °C, the G band intensity significantly diminished by two orders of magnitude. One can note that the decrease in G band intensity stems from the decrease in the amount of nanocarbons. According to the previous TGA studies,^49–52^ nanocarbons start to desorb from the sample at around 400 °C. However, it was reported that almost 33% of nanocarbons remains on the metal nanoparticles after annealing at 500 °C.^54^ Thus, it is difficult to explain the drastic decrease in G band intensity at 395 °C solely by the decrease in the amount of nanocarbons.

We consider that the decrease in G band intensity at 395 °C is mainly attributed to the deactivation of the SERS effect as a result of the sintering of AgNCs. This temperature is significantly lower than the melting points of bulk Ag and a single AgNC.^55^ It has been reported that 10–100 nm-sized aggregates of AgNPs, *i.e.*, Ag ink paste, coalesced at 200–250 °C.^48,56–60^ This coalescence of AgNPs occurs at a temperature significantly lower than the melting points of corresponding sizes of AgNPs^61,62^ and is widely known to be a result of sintering. According to the previous photoabsorption spectroscopy study, the irradiation of a 532 nm laser to AgNCs induces the excitation of the single-particle mode of AgNCs' LSP.^27^ This indicates that hot spots for SERS are preserved as long as the individual AgNC structures are maintained. However, when the sintering of AgNCs occurs, the G band intensity is expected to be suppressed because of the absence of the hot spots of AgNCs.^63^ As seen in the temperature dependence of the AgNC height (Fig. 5), a slight decrease in AgNC height was confirmed at 222 °C. At the same time, the SEM image (Fig. 4(b)) suggests that the AgNC gaps were filled with NPs. This is probably due to the gap narrowing caused by the lateral deformation of AgNCs, leading to the emergence of nanocarbons from the AgNC gaps. Since the sublimation of AgNCs does not occur at 222 °C, the height change observed here is not caused by the desorption of Ag. If we assume the isotropic deformation of AgNCs along lateral directions, the height decrease of 7.4 nm (17.4 nm) corresponds to the width increase of 3.9 nm (10.0 nm) at 265 °C (395 °C), respectively. Note that the width increase of 10.0 nm for each AgNC at 395 °C suggests the complete filling of the AgNC gap (10 nm). Therefore, we conclude that the sintering of AgNCs occurred between 265 °C and 395 °C. We presume that in the initial stage of sample annealing, nanocarbons were formed on the AgNC surface. Some nanocarbons aggregated and moved to the AgNC–AgNC gap upon further annealing. Fig. 6(b) shows plots of the D/G ratio against annealing temperature. The Raman intensity of the D band normalized by that of the G band is referred to as the D/G ratio, which has been used as an index of defect density in nanocarbons.^47^ The D/G ratio initially dropped from 179 °C to 222 °C; however, it subsequently rose at 482 °C. This result indicates that defective nanocarbons were formed by annealing.

It is considered that nanocarbons formed from PVP no longer exist in the AgNC gaps at 395 °C since the sintering requires the formation of metal–metal contacts, as was suggested by previous researchers.^56,57,59,64–66^ Structural changes of AgNCs and the thermal desorption of nanocarbons induce the elimination of nanocarbons from the AgNC nanogap at 395 °C, followed by the exposure of the clean Ag surface and finally the sintering of AgNCs. Such elimination of the covering molecules has been similarly observed for Ag nanopillars.^48^ Note that in the case of spherical NPs, the sintering and shape change occur after the desorption of the covering molecules.^56,59^ This is because spherical NPs have good thermal stability; thus, the desorption of the covering molecules occurs below the deformation temperature of NPs.

**Fig. 7:**
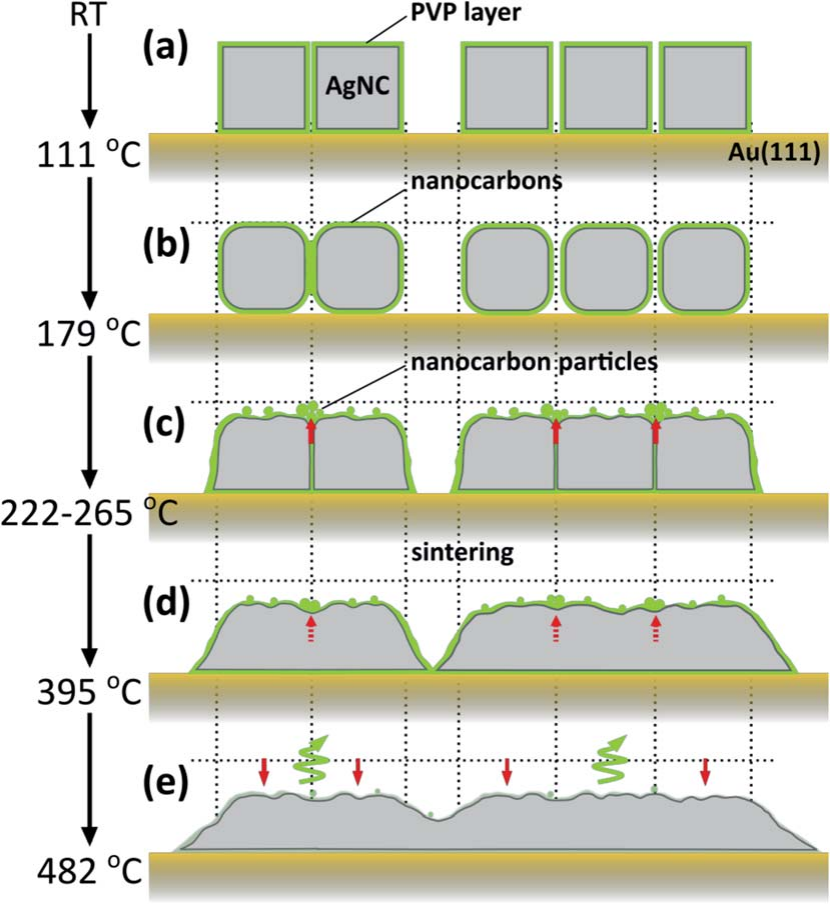
Schematic illustrations of the thermally induced structural and chemical changes of PVP/AgNCs/Au (111). (a) AgNCs at RT and after annealing at 111 °C. Up to 111 °C, the structures of individual AgNCs and the array are preserved. (b) Up to 179 °C, the corners of AgNCs become rounded. PVP begins to decompose into nanocarbons. Some AgNCs no longer preserved the gap structure. (c) Up to 222–265 °C, nanocarbon particles emerge from the AgNC gap owing to gap narrowing. (d) Up to 395 °C, nanocarbons are completely eliminated from gaps and the sintering of AgNCs occurs. The decomposition and desorption of nanocarbons start. (e) Up to 482 °C, the AgNC grains collapse and the AgNC height decreases. Simultaneously, the decomposition and desorption of nanocarbons proceed.

Fig. 7 shows schematic illustrations of the thermally induced structural and chemical changes of PVP/AgNCs/Au(111). Up to 111 °C, although individual and array structures of AgNCs are preserved, some of the pyrrolidone rings are opened. When heated to 179 °C, the appearance of D and G bands in the Raman spectrum indicates that PVP begins to decompose into nanocarbons. In addition, the corners of AgNCs become rounded. When heated to 265 °C, the thermal decomposition of PVP proceeds, so that the amount of nanocarbons increases; this is also seen in the AgNC gaps. Here, nanocarbon particles emerge from the AgNC gaps owing to gap narrowing. Such gap narrowing occurs *via* the lateral expansion of AgNCs accompanying the decrease in AgNC height. When heated to 395 °C, significant decreases in D and G band intensities suggest the deactivation of the SERS effect by the decrease of the LSP-induced electric filed enhancement, which is caused by the sintering of AgNCs. The higher the annealing temperature, the greater the decrease in AgNC height, which ends in the collapse of AgNC grains. Simultaneously, the decomposition and desorption of nanocarbons proceed, and nanocarbons become more defective, as seen in the increase in D/G ratio.

**Fig. 8:**
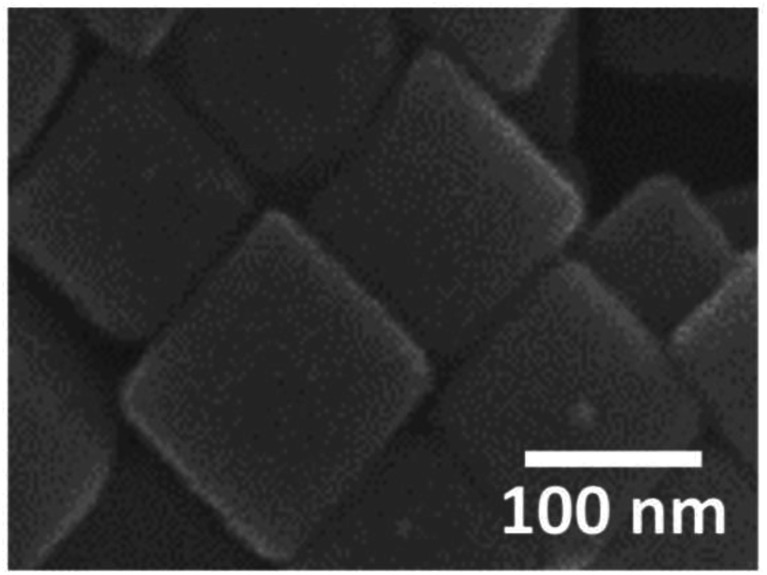
SEM image obtained after irradiating an intense laser (*λ* = 532 nm, 4 mW μm^−2^) on PVP/AgNC/Au (111) for 4 s. *V*_Acc_ was set at 10 kV.

Finally, we briefly present the results related to the photoinduced chemical changes of PVP/AgNCs/Au(111). Fig. 8 shows an SEM image obtained after the irradiation of an intense laser (*λ* = 532 nm, 4 mW μm^−2^) to the sample surface for 4 s (2 s × 2 times). We found that individual AgNCs are intact after the irradiation of the intense laser. This indicates that the intense laser used in this study would not have sufficient power to induce the structural changes of AgNCs. However, obvious changes were confirmed in the Raman spectrum after the irradiation of the intense laser, as represented by the appearance of D and G bands, as shown in Fig. 3(h). Interestingly, the PVP peaks in the Raman spectrum remain intact even though the same power of the 785 nm laser was irradiated. The dependence of wavelength on the reaction observed here strongly suggests that PVP was converted to nanocarbons not thermally but photo-chemically. We confirmed that the photoreaction of PVP did not proceed without AgNCs, *i.e.*, PVP/Au(111), indicating that LSP-induced electric field enhancement at AgNCs is involved in this reaction process. D and G band intensities obtained after photoreaction (Fig. 3(h)) are approximately four times larger than those obtained after annealing (Fig. 3(d)). This is probably because AgNCs remained intact after laser irradiation; hence, the SERS effect is dominant in the Raman spectrum. In addition, after the photoreaction, nanocarbon particles were not seen on the AgNC surfaces. This is clear evidence that the formation of nanocarbon particles is driven thermally.

## 4. Conclusions

Thermally and photoinduced structural changes of a PVP-covered AgNC array on Au(111) were investigated. Owing to the LB method, AgNCs are aligned in the face-to-face configuration, forming a two-dimensional monolayer. In the Raman spectrum obtained at room temperature, sharp peaks assigned to vibrational modes of PVP were observed as a result of the SERS effect of the AgNCs. When annealing AgNCs up to 179 °C, the alignment of AgNCs remained unchanged while the corners of individual AgNCs were rounded. Simultaneously, PVP was thermally converted to nanocarbons. The decreases in G band intensity and AgNC height indicated that the sintering of AgNCs occurred between 265 °C and 395 °C. Structural changes of AgNCs and the thermal desorption of nanocarbons lead to the elimination of nanocarbons from the AgNC nanogap, followed by the exposure of the clean Ag surface and finally the sintering of AgNCs. By irradiating an intense laser on the AgNC surface, we also confirmed the transformation of PVP to nanocarbons on AgNCs without the deformation of AgNCs. The strong peaks of both D and G bands appearing after the photoreaction are due to the SERS effect of AgNCs.

## Conflicts of interest

There are no conflicts of interest to declare.

## Supplementary Material
